# Revisiting the L-Dopa Response as a Predictor of Motor Outcomes After Deep Brain Stimulation in Parkinson’s Disease

**DOI:** 10.3389/fnhum.2021.604433

**Published:** 2021-02-04

**Authors:** Zhengyu Lin, Xiaoxiao Zhang, Linbin Wang, Yingying Zhang, Haiyan Zhou, Qingfang Sun, Bomin Sun, Peng Huang, Dianyou Li

**Affiliations:** ^1^Department of Neurosurgery, Ruijin Hospital, Shanghai Jiao Tong University School of Medicine, Shanghai, China; ^2^Center for Functional Neurosurgery, Ruijin Hospital, Shanghai Jiao Tong University School of Medicine, Shanghai, China; ^3^Department of Neurology, Ruijin Hospital, Shanghai Jiao Tong University School of Medicine, Shanghai, China

**Keywords:** Parkinson’s disease, L-dopa challenge test, deep brain stimulation, globus pallidus interna, subthalamic nucleus

## Abstract

**Objective:** To investigate the correlation between preoperative response to the L-dopa challenge test and efficacy of deep brain stimulation (DBS) on motor function in Parkinson’s disease (PD).

**Methods:** We retrospectively reviewed the data of 38 patients with idiopathic PD who underwent DBS surgery with a median follow-up duration of 7 months. Twenty underwent bilateral globus pallidus interna (GPi) DBS, and 18 underwent bilateral subthalamic nucleus (STN) DBS. The Movement Disorder Society Unified Parkinson Disease Rating Scale-Motor Part (MDS UPDRS-III) was assessed before surgery and at the last follow-up in different medication and stimulation conditions, respectively.

**Results:** Pearson’s correlation analysis revealed a positive correlation between preoperative L-dopa challenge responsiveness and GPi-DBS responsiveness on the total score (*R*^2^ = 0.283, *p* = 0.016) but not on the non-tremor total score (*R*^2^ = 0.158, *p* = 0.083) of MDS UPDRS-III. Such correlation remained significant (*R*^2^′ = 0.332, *p* = 0.010) after controlling for age at the time of surgery as confounding factor by partial correlation analysis. The preoperative L-dopa challenge responsiveness was significantly correlated with the tremor-controlling outcome of GPi-DBS (*R*^2^ = 0.390, *p* = 0.003). In contrast, we found a positive correlation between preoperative L-dopa challenge responsiveness and STN-DBS responsiveness on the non-tremor total score (*R*^2^ = 0.290, *p* = 0.021), but not on the total score (*R*^2^ = 0.130, *p* = 0.141) of MDS UPDRS-III. The partial correlation analysis further demonstrated that the predictive value of preoperative L-dopa challenge responsiveness on the non-tremor motor outcome of STN-DBS was eliminated (*R*^2^′ = 0.120, *p* = 0.174) after controlling for age at the time of surgery as confounding factor.

**Interpretation:** The short-term predictive value of preoperative response to the L-dopa challenge test for the motor outcome of GPi-DBS in PD was systematically described. Our findings suggest: (1) a solid therapeutic effect of GPi-DBS in treating L-dopa-responsive tremors; (2) a negative effect of age at the time of surgery on motor outcomes of STN-DBS, (3) a possible preference of STN- to GPi-DBS in L-dopa-resistant tremor control, and (4) a possible preference of GPi- to STN-DBS in elderly PD patients who have a satisfactory dopamine response.

## Introduction

Parkinson’s disease (PD) is the second most common neurodegenerative disorder characterized by striatal dopamine deficiency, resulting from a selective neuronal loss in the substantia nigra (Kalia and Lang, 2015). The cardinal motor dysfunctions of PD include bradykinesia, tremor, rigidity, and axial symptoms (Armstrong and Okun, 2020). Deep brain stimulation (DBS) is a well-established neurosurgical treatment for motor fluctuations and dyskinesia in patients with advanced PD (Li et al., 2013; Zhou et al., 2019). The subthalamic nucleus (STN) and globus pallidus interna (GPi) are most studied for DBS surgery in PD. Randomized clinical trials have reported similar, consistent, and notable motor benefits with subtle differences between these two areas (Ramirez-Zamora and Ostrem, 2018).

Satisfactory L-dopa responsiveness, a critical component of the diagnosis of idiopathic PD (Rizzo et al., 2016; Armstrong and Okun, 2020), has been the widely accepted criterion for DBS surgery patient selection (Pollak, 2013). Nonetheless, the predictive value of preoperative L-dopa responsiveness for the outcome of DBS remains controversial. Charles et al. (2002) and Welter et al. (2002) reported the value of preoperative L-dopa responsiveness as a predictor of STN-DBS efficacy on motor function in PD during the 3- and 6-month follow-up, respectively. However, Piboolnurak et al. (2007) argued that the magnitude of the preoperative response to L-dopa was limited in predicting the benefits of STN-DBS at the 3- and 5-year follow-up. Zaidel et al. (2010) also had indicated the methodological drawbacks of previous studies and the need for the reassessment of such strong correlations. Moreover, most previous studies only enrolled patients undergoing STN-DBS, while the value of preoperative L-dopa responsiveness as a predictor for GPi-DBS responsiveness has not been adequately studied.

In this retrospective study, we aimed to describe the value of preoperative L-dopa responsiveness following the L-dopa challenge test for predicting motor outcomes of GPi-DBS in PD. We also focused on revisiting the predictive value of preoperative L-dopa challenge responsiveness for the effects of STN-DBS. Propositions for target selections were further proposed based on this study.

## Materials and Methods

### Participants

We retrospectively studied 38 patients with PD who underwent simultaneous bilateral GPi- (20 patients) or STN-DBS (18 patients) from November 2017 to July 2019 at the Center for Functional Neurosurgery, Ruijin Hospital (Shanghai, China). The Ethical Committee of Ruijin Hospital approved the study protocol. All patients provided written informed consent for surgery and participation in the follow-up. The authors have no ethical conflicts to disclose. The GPi group was predominantly selected before mid-2018, while the STN group was selected after mid-2018 during the study period. The inclusion criteria for DBS surgery were as follows: (1) diagnosis of idiopathic PD by an experienced movement disorder specialist based on the UK PD Brain Bank Criteria (Hughes et al., 1992); (2) response to L-dopa following a preoperative L-dopa challenge test (see below); (3) experiences of disabling motor fluctuations, wearing-off phenomena, or dyskinesia; (4) consent to undergo DBS surgery; and (5) accommodation of regular postoperative programming and follow-ups. The exclusion criteria were as follows: (1) contraindication for neurosurgery, (2) comorbidities such as dementia or severe neuropsychiatric disorders, (3) comorbidities including other organic cerebral abnormalities, and (4) contraindication for high-field magnetic resonance imaging (MRI).

### Clinical Assessment

We collected the clinical information (e.g., demographics and medical history) of all patients before surgery. The L-dopa equivalent dose (LED) in the preoperative medication regimen was calculated according to Tomlinson et al. (2010). Motor function was evaluated preoperatively using the Movement Disorder Society Unified Parkinson Disease Rating Scale-Motor Part (MDS UPDRS-III) (Goetz et al., 2008) and was scored in both off (MedOff) and on antiparkinsonian medication (MedOn) conditions. Similarly, MDS UPDRS-III was assessed at the last follow-up in both off-medication/off-stimulation (MedOff/StimOff) and off-medication/on-stimulation conditions (MedOff/StimOn).

Patients were evaluated in a fasting state and after stopping antiparkinsonian medication for at least 12 h (usually overnight) for the MedOff condition. A single supra-threshold dose of L-dopa (the usual effective dose taken in the morning ×1.5) (Pollak, 2013) was administered subsequently for the MedOn condition, in which the patient and the investigator agreed that the best functional benefits were achieved (Defer et al., 1999). A mild degree of dyskinesia was observed in a few cases following the L-dopa challenge test, and had insignificant impact on motor function evaluation. Similarly, at the last follow-up, the evaluation for the MedOff/StimOff condition was performed following overnight dopaminergic medication cessation and turning stimulation off for 0.5 h. For the MedOff/StimOn condition, evaluations were performed 1 h after restarting stimulation. The motor examination was performed and videotaped by one evaluator and independently scored (excepted for the rigidity item) by one experienced movement disorder specialist; the rigidity subscore was directly scored by the evaluator during the examination. Both of these two raters were blinded to medication and stimulator statuses.

### Levodopa Responsiveness

The L-dopa responsiveness for motor functions, which refers to the relative improvement in the MDS UPDRS-III score after the L-dopa challenge test, was calculated using Eq. 1:


(1) $$\text{Preoperative }response\;to\;L‐\text{dopa }\left(\%\right)=\;\frac{\text{MedOff}-\text{MedOn}}{\text{MedOff}}\times 100\%$$

The cut-off value for preoperative L-dopa responders and non-responders was 24% (Merello et al., 2011). The postoperative DBS responsiveness, adapted from Zaidel et al. (2010), was defined as follows:


(2) $$\text{Postoperative }response\;to\;DBS\;\left(\text{\%}\right)=\;\frac{\text{MedOff/StimOff}-\text{MedOff/StimOn}}{\text{MedOff/StimOff}}\times 100\%.$$

A series of parkinsonian symptoms were analyzed individually: (1) rigidity (item 3.3); (2) tremor (items 3.15–3.18); (3) bradykinesia (items 3.2, 3.4–3.8, and 3.14); and (4) axial symptoms (items 3.1 and 3.9–3.13). The non-tremor total score of MDS UPDRS-III was calculated as the sum of subscores of rigidity, bradykinesia, and axial symptoms.

### Surgical Technique and Planning

All patients underwent preoperative MRI (3.0 T) before surgery. On the day of surgery, a Leksell stereotactic frame was mounted followed by a head CT scan. The specific target coordinates and trajectory were defined using the SurgiPlan system (Elekta AB, Sweden) after the coregistration of MRI-CT images, targeting the dorsolateral STN and the posterior GPi. All surgical procedures were performed under general anesthesia. The implantable pulse generator (IPG) was placed subclavicularly and was connected with electrodes via subcutaneous wires. Postoperative imaging was performed to confirm the satisfactory placement of DBS leads.

Implantable pulse generator programming was initiated on the following day, and parameters including voltage, pulse-width, and frequency were optimized within the first 3 months of surgery and adjusted by two experienced movement specialists (HZ and DL) who referred to the Chinese standardized protocol (Chen et al., 2018). Briefly, monopolar stimulation was preferred during the initial programming sessions. The contacts were individually tested to inspect patients’ motor response and assess side effects. Generally, the initial parameters were set to monopolar mode, with a pulse-width of ∼60 μs and a frequency of ∼130 Hz, and a stepwise increase in amplitude according to the patient’s response. A bipolar mode was preferred when the stimulating response was limited by side effects under monopolar settings (Chen et al., 2018). The mean stimulating parameters at the last follow-up are provided in Supplementary Table 1.

### Data Analyses

Statistical analyses were performed using SPSS software (Version 23.0. Amonk, NY, United States: IBM Corp.). Continuous variables were presented as means ± standard deviations with or without a range. The categorical variables were expressed as frequencies (%). We used Student’s *t*-test or Wilcoxon signed-rank test to compare between the preoperative and postoperative baselines (i.e., MedOff and MedOff/StimOff, respectively), and between the MedOff/StimOff and MedOff/StimOn conditions. Pearson’s correlation analysis was performed to determine the relevance of the relationship between preoperative L-dopa challenge responsiveness and postoperative DBS responsiveness. The potential contribution of baseline characteristics (e.g., age at the time of surgery, disease duration, and LED) was also evaluated by Pearson’s correlation. Baseline factors that were significantly correlated with the motor outcomes of DBS were then entered into the partial correlation analysis as confounding factors. A *p*-value < 0.05 was considered statistically significant.

## Results

### Demographic and Clinical Information

A total of 38 patients (25 men and 13 women) were included in the analysis, of whom 20 were implanted with GPi and 18 with STN. The mean disease duration was 10.2 ± 3.8 years, and the mean age at the time of surgery was 58.8 ± 11.2 years. The mean Hoehn–Yahr stage was 3.2 ± 0.9 and 2.6 ± 0.8 in the off- and on-medication states, respectively. The median follow-up duration was 7 [interquartile range (IQR): 6–12] months. Dyskinesia was reported in 53% (20/38) of patients. The LED was 747.0 ± 302.7 mg/day. The demographic characteristics are listed in Table 1.

**TABLE 1:** Patients’ demographics and clinical information.

Characteristics	Total	GPi	STN
	(*N* = 38)	(*N* = 20)	(*N* = 18)
Male	25	12	13
Female	13	8	5
Age at PD onset (year)	48.5 ± 11.1	52.0 ± 8.0	45.7 ± 11.4
Age at the time of surgery (year)	58.8 ± 11.2	62.0 ± 9.1	55.9 ± 12.3
Disease duration* (year)	10.2 ± 3.8	10.0 ± 3.9	10.3 ± 3.2
**Hoehn–Yahr stage**			
Off-medication	3.2 ± 0.9	3.3 ± 0.9	3.2 ± 0.9
On-medication	2.6 ± 0.8	2.7 ± 0.9	2.4 ± 0.8
Last follow-up (month)	7 (6–12)	12 (6–13.5)	6 (6–8)
Presence of dyskinesia	20 (53%)	9 (45%)	11 (61%)
L-dopa equivalent dose (mg/day)	747.0 ± 302.7	761.4 ± 247.9	731.8 ± 360.5

Continuous variables are presented as means ± SDs or medians with interquartile ranges. Categorical variables are presented as frequencies (%). *Defined as the amount of time since self-reported onset of motor symptoms. GPi, globus pallidus interna; STN, subthalamus nucleus.

### Pre- and Postoperative Baselines of MDS UPDRS-III Total Score and Subscores

A comparison of the total MDS UPDRS-III scores between the MedOff and the MedOff/StimOff condition showed no statistical significance in both GPi (46.2 ± 12.4 versus 48.4 ± 10.1, *p* = 0.371) and STN groups (61.6 ± 15.8 versus 60.5 ± 13.6, *p* = 0.761). Similarly, there was no significant difference of the total non-tremor MDS UPDRS-III scores between the MedOff and MedOff/StimOff states in both GPi (40.4 ± 12.7 versus 44.0 ± 9.2, *p* = 0.132) and STN (50.5 ± 13.8 versus 52.5 ± 13.0, *p* = 0.478) groups. Regarding MDS UPDRS-III subscores, the tremor score was significantly lower in the MedOff/StimOff state (8.0 ± 5.5) than that in the MedOff state (11.1 ± 6.4) (*p* = 0.021) in STN group. This tendency was also observed in GPi group, although it was not statistically significant (4.4 ± 5.4 versus 5.8 ± 5.7, *p* = 0.107). Moreover, in GPi group, the bradykinesia score in the MedOff/StimOff condition (24.9 ± 6.3) was significantly higher compared with the MedOff condition (20.4 ± 6.1) (*p* = 0.008). A similar trend was observed in the STN group (29.2 ± 8.2 versus 26.2 ± 8.5, *p* = 0.058). There were no significant differences of rigidity and axial symptom subscores between the MedOff and MedOff/StimOff states in both GPi and STN groups (Table 2).

**TABLE 2:** Efficacy of globus pallidus interna or subthalamic nucleus deep brain stimulation on off-medication MDS UPDRS-III total score and subscores.

MDS UPDRS-III	GPi			STN		
	(*N* = 20)	*p*_1_	*p*_2_	(*N* = 18)	*p*_1_	*p*_2_
**Total score**						
MedOff	46.2 ± 12.4	0.371		61.6 ± 15.8	0.761	
MedOff/StimOff	48.4 ± 10.1		1.9 × 10^–5^*	60.5 ± 13.6		4.0 × 10^–6^*
MedOff/StimOn	35.7 ± 12.9			36.9 ± 14.4		
**Non-tremor total score**						
MedOff	40.4 ± 12.7	0.132		50.5 ± 13.8	0.478	
MedOff/StimOff	44.0 ± 9.2		3.0 × 10^–6^*	52.5 ± 13.0		1.7 × 10^–4^*
MedOff/StimOn	35.2 ± 12.6			35.1 ± 13.9		
**Rigidity**						
MedOff	10.7 ± 4.9	0.926		13.2 ± 4.0	0.829	
MedOff/StimOff	10.6 ± 3.3		0.029*	13.1 ± 2.8		9.7 × 10^–4^*
MedOff/StimOn	8.8 ± 3.5			7.1 ± 4.4		
**Tremor**						
MedOff	5.8 ± 5.7	0.107		11.1 ± 6.4	0.021*	
MedOff/StimOff	4.4 ± 5.4		4.2 × 10^–4^*	8.0 ± 5.5		6.4 × 10^–4^*
MedOff/StimOn	0.5 ± 1.1			1.8 ± 3.2		
**Bradykinesia**						
MedOff	20.4 ± 6.1	0.008*		26.2 ± 8.5	0.058	
MedOff/StimOff	24.9 ± 6.3		4.0 × 10^–6^*	29.2 ± 8.2		2.5 × 10^–3^*
MedOff/StimOn	19.3 ± 8.1			21.1 ± 8.8		
**Axial symptoms**						
MedOff	9.4 ± 4.2	0.388		11.1 ± 4.2	0.458	
MedOff/StimOff	8.6 ± 3.8		0.001*	10.3 ± 4.7		2.1 × 10^–4^*
MedOff/StimOn	7.0 ± 3.7			6.9 ± 3.9		

Values are presented as means ± SDs. *p*_1_ indicates the statistical difference of the MDS UPDRS-III score and subscores between the MedOff and MedOff/StimOff states. *p*_2_ indicates the statistical difference of the MDS UPDRS-III score and subscores between the MedOff/StimOff and MedOff/StimOn states. *p-value < 0.05. MDS UPDRS-III, Movement Disorder Society Unified Parkinson’s Disease Rating Scale-Motor Part; GPi, globus pallidus interna; STN, subthalamus nucleus; MedOff, preoperative off-medication state; MedOff/StimOff, postoperative off-medication/off-stimulation state; MedOff/StimOff, postoperative off-medication/on-stimulation state.

### L-Dopa Responsiveness on Motor Functions

Overall, the percentage improvement in the MDS UPDRS-III total score following the L-dopa challenge test before surgery was 50.8 ± 16.3 and 50.9 ± 12.1% for the GPi and STN groups, respectively. Similarly, the L-dopa challenge responsiveness on the total non-tremor MDS UPDRS-III scores, and the rigidity, tremor, bradykinesia, and axial symptom subscores for the GPi (47.0 ± 17.5, 51.1 ± 25.4, 56.9 ± 44.3, 45.0 ± 22.5, and 48.6 ± 24.7%, respectively) and STN groups (44.9 ± 12.8, 44.1 ± 24.8, 79.1 ± 30.0, 41.8 ± 14.3, and 53.8 ± 20.8%, respectively) are presented in Table 3.

**TABLE 3:** Preoperative L-dopa responsiveness and deep brain stimulation responsiveness at the last follow-up on the MDS UPDRS-III score and subscores.

Scale	GPi	STN
	(*N* = 20)	(*N* = 18)
**MDS UPDRS-III**		
**Total score**		
L-dopa responsiveness	50.8 ± 16.3% (28–82%)	50.9 ± 12.1% (30–77%)
DBS responsiveness	26.7 ± 19.0% (6–71%)	42.5 ± 15.5% (25–72%)
**Non-tremor total score**		
L-dopa responsiveness	47.0 ± 17.5% (22–82%)	44.9 ± 12.8% (21–73%)
DBS responsiveness	21.8 ± 16.6% (2–53%)	37.2 ± 15.6% (18–70%)
**Rigidity**		
L-dopa responsiveness	51.1 ± 25.4% (8–100%)	44.1 ± 24.8% (−10 to 100%)
DBS responsiveness	13.6 ± 28.5% (−27 to 69%)	56.0 ± 16.8% (28–82%)
**Tremor**		
L-dopa responsiveness	56.9 ± 44.3% (0–100%)	79.1 ± 30.0% (0–100%)
DBS responsiveness	73.4 ± 40.2% (0–100%)	68.2 ± 48.4% (0–100%)
**Bradykinesia**		
L-dopa responsiveness	45.0 ± 22.5% (0–86%)	41.8 ± 14.3% (−13 to 67%)
DBS responsiveness	25.1 ± 18.3% (0–61%)	30.2 ± 20.8% (−11 to 65%)
**Axial symptoms**		
L-dopa responsiveness	48.6 ± 24.7% (0–100%)	53.8 ± 20.8% (25–100%)
DBS responsiveness	15.4 ± 29.2% (−50%–67%)	34.0 ± 26.2% (0–100%)

*Values are presented as means ± SDs (ranges). DBS, deep brain stimulation; MDS UPDRS-III, Movement Disorder Society Unified Parkinson’s Disease Rating Scale-Motor Part; GPi, globus pallidus interna; STN, subthalamus nucleus.*

### DBS Responsiveness on Motor Functions

The total MDS UPDRS-III score and tremor score demonstrated a significant improvement in the MedOff/StimOn condition compared with that in the MedOff/StimOff condition in patients with GPi-DBS. The total MDS UPDRS-III score improved by 26.7 ± 19.0% from 48.4 ± 10.1 at postoperative baseline to 35.7 ± 12.9 (*p* = 1.9 × 10^–5^). Similarly, compared to the MedOff/StimOff state, the total non-tremor MDS UPDRS-III score as well as the rigidity, tremor, bradykinesia, and axial symptom subscores all showed significant amelioration in the MedOff/StimOn state in GPi group (*p* = 3.0 × 10^–6^, 0.029, 4.2 × 10^–4^, 4.0 × 10^–6^, and 0.001, respectively). For STN group, the total MDS UPDRS-III score improved by 42.5 ± 15.5% from 60.5 ± 13.6 at postoperative baseline to 36.9 ± 14.4 (*p* = 4.0 × 10^–6^). The total non-tremor MDS UPDRS-III score and the subscores of rigidity, tremor, bradykinesia, and axial symptoms were significantly lower in the MedOff/StimOn condition than in the MedOff/StimOff condition (*p* = 1.7 × 10^–4^, 9.7 × 10^–4^, 6.4 × 10^–4^, 2.5 × 10^–3^, and 2.1 × 10^–4^, respectively) (Tables 2, 3).

### Correlation Between L-Dopa Challenge Responsiveness and DBS Responsiveness on MDS UPDRS-III Score and Subscores

**FIGURE 1:**
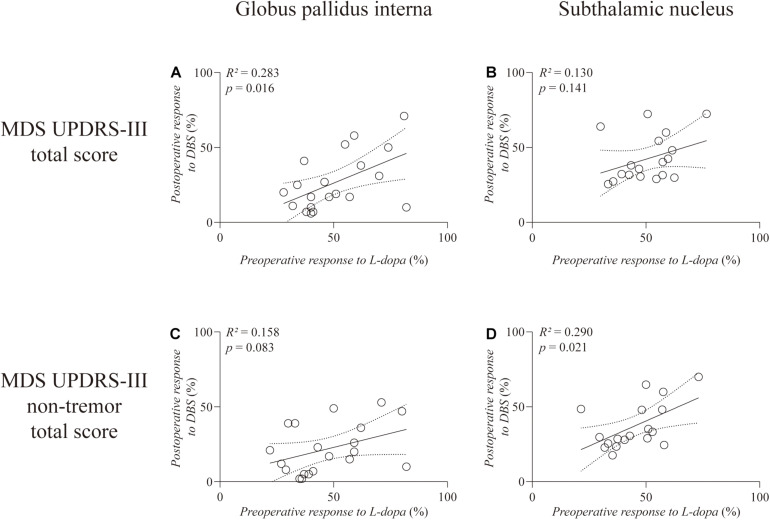
Linear regression between preoperative L-dopa challenge responsiveness and DBS responsiveness on the total score **(A,B)** and the non-tremor total score **(C,D)** of MDS UPDRS-III. The left column is the GPi group and the right column is the STN group. Individual data are represented as black circles. The 95% confidence interval for the linear regression is shown as dotted lines.

As illustrated in Figure 1, correlation analysis showed a significant and positive correlation between the preoperative response to L-dopa and response to GPi-DBS on the total MDS UPDRS-III score (*R*^2^ = 0.283, *p* = 0.016) (Figure 1A). Such correlation disappeared when the tremor subscore was excluded (*R*^2^ = 0.158, *p* = 0.083) (Figure 1C). Regarding patients with STN-DBS, there was no significant correlation between preoperative L-dopa responsiveness and STN-DBS responsiveness on the total MDS UPDRS-III score (*R*^2^ = 0.130, *p* = 0.141) (Figure 1B). However, STN-DBS responsiveness was positively correlated with preoperative L-dopa challenge responsiveness with statistical significance (*R*^2^ = 0.290, *p* = 0.021) on the non-tremor total score of MDS UPDRS-III (Figure 1D).

**FIGURE 2:**
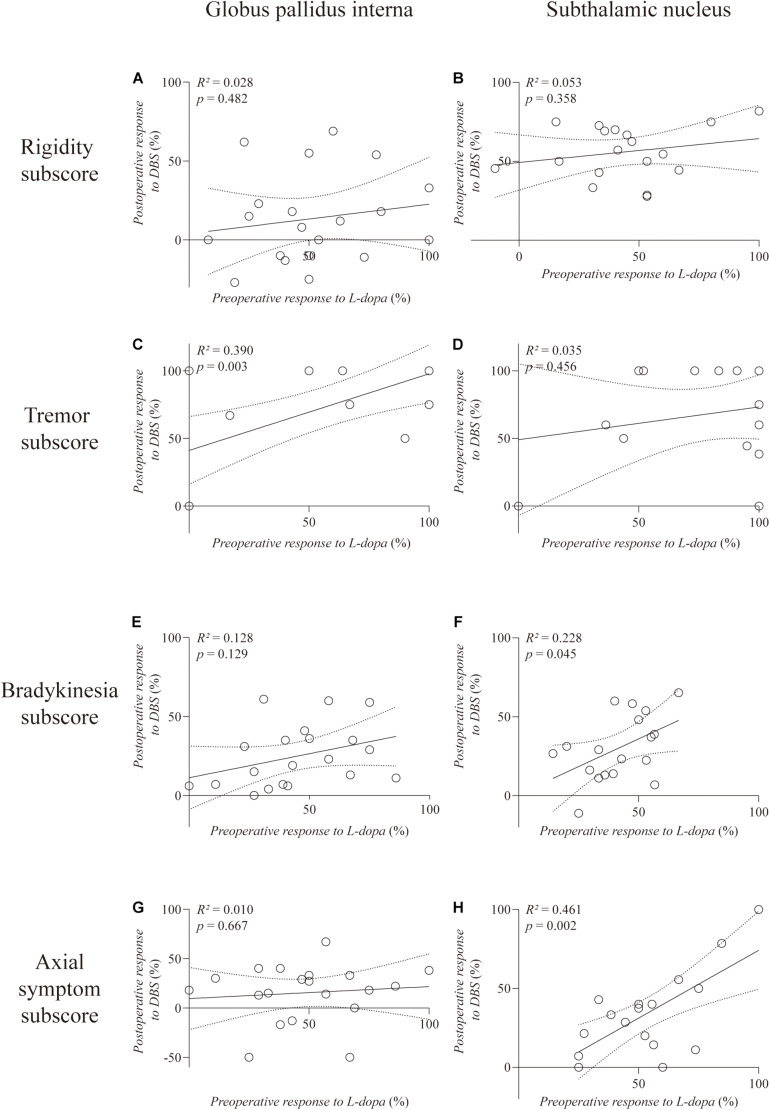
Linear regression between preoperative L-dopa challenge responsiveness and DBS responsiveness on the MDS UPDRS-III subscores. The left column is the GPi group and the right column is the STN group. The rigidity **(A,B)**, tremor **(C,D)**, bradykinesia **(E,F)**, and axial symptom **(G,H)** subscores are, respectively, plotted. Individual data are represented as black circles. The 95% confidence interval for the linear regression is shown as dotted lines.

Analysis of the MDS UPDRS-III subscores (Figure 2) revealed a significantly positive correlation between the preoperative response to L-dopa and response to GPi-DBS on the tremor subscore (*R*^2^ = 0.390, *p* = 0.003) (Figure 2C). However, the response to STN-DBS was not correlated with preoperative L-dopa challenge responsiveness on the tremor subscore (*R*^2^ = 0.035, *p* = 0.456). Moreover, STN-DBS responsiveness was positively correlated with preoperative L-dopa challenge responsiveness, with statistical significance regarding the subscores of bradykinesia (*R*^2^ = 0.228, *p* = 0.045) and axial symptoms (*R*^2^ = 0.461, *p* = 0.002), respectively (Figures 2F,H). No correlation was observed in the GPi and STN groups on the rigidity subscore (*R*^2^ = 0.028, *p* = 0.482; *R*^2^ = 0.053, *p* = 0.358, respectively).

One baseline factor, age at the time of surgery, was entered into the partial correlation analysis as significant negative correlations with STN-DBS responsiveness on the total and non-tremor total scores of MDS UPDRS-III were observed (*R*^2^ = 0.228, *p* = 0.045; *R*^2^ = 0.263, *p* = 0.030, respectively). There were no significant correlations between the other two baseline factors (i.e., disease duration and LED) and DBS responsiveness on the total score and non-tremor total score of MDS UPDRS-III in the GPi and STN groups. Partial correlation analysis further revealed that the correlation remained significant between the preoperative response to L-dopa and the response to GPi-DBS on the total MDS UPDRS-III score (*R*^2^′ = 0.332, *p* = 0.010), while the significant correlation disappeared between the preoperative L-dopa challenge responsiveness and STN-DBS responsiveness on the non-tremor total score of MDS UPDRS-III (*R*^2^′ = 0.120, *p* = 0.174).

## Discussion

This retrospective study assessed the short-term predictive value of preoperative L-dopa challenge responsiveness for the improvement in the MDS UPDRS-III for PD patients undergoing STN- or GPi-DBS. We found that the value of the preoperative response to the L-dopa challenge test for predicting the short-term motor outcome of STN-DBS was limited, as the significant negative correlation between the age at the time of surgery and the motor outcome of STN-DBS may considerably interfere with the predictive value of preoperative L-dopa challenge responsiveness. We also demonstrated the preoperative response to the L-dopa challenge test as a predictor of motor outcomes, especially the tremor-controlling effects for GPi-DBS. To the best of our knowledge, despite the short-term follow-up, our work is the first to systematically describe the predictive value of preoperative L-dopa responsiveness for GPi-DBS motor responsiveness.

Unlike some previous studies (Charles et al., 2002; Welter et al., 2002; Pahwa et al., 2005; Su et al., 2017), we have calculated the benefits of stimulation under the MedOff/StimOn state, by comparing the respective MDS UPDRS-III score and subscores in the MedOff/StimOff and MedOff/StimOn states, instead of comparing them with the preoperative MedOff scores. The advantage of using MedOff/StimOn state as the baseline for stimulation responsiveness calculation is that the disease progression, as well as the underlying insertional microlesion effects (Mann et al., 2009), will be taken into consideration. Moreover, we used relative rather than absolute changes for comparing pre- and postoperative improvements, as proposed by Zaidel et al. (2010). However, unlike Zaidel et al. (2010) who used on-medication/on-stimulation scores for evaluating the DBS responsiveness, we preferred to perform calculations in the postoperative off-medication state so that only the benefits of stimulation, rather than a combination of stimulation and medication, would be taken into account. Furthermore, the use of the postoperative on-medication state for correlation analysis would have been confusing since the pre- and postoperative medication dosages for motor function evaluation were different (i.e., preoperative L-dopa challenge test versus regular postoperative morning dose).

The origin of tremor in PD remains to be elucidated. Clinically, L-dopa replacement therapy can effectively treat bradykinesia and rigidity, while the effect on resting tremor is unpredictable and has strong inter-individual variations (Koller, 1986; Koller and Hubble, 1990). Tremors in PD are usually characterized by a combination of L-dopa-responsive and L-dopa-resistant features (Zach et al., 2020), suggesting an involvement non-dopaminergic neural circuits in tremor pathophysiology (Nonnekes et al., 2016; Dirkx et al., 2019). For instance, reduced raphe serotonin function is associated with the severity of parkinsonian resting tremors and poor response to L-dopa (Qamhawi et al., 2015; Pasquini et al., 2018). Enhanced noradrenergic function is correlated with increased tremor intensity in some patients with PD during cognitive stress (Isaias et al., 2012; Zach et al., 2017). We observed that the baseline tremor score in the MedOff/StimOff state was significantly lower than that in the preoperative MedOff state in the STN group; a similar tendency was also observed in the GPi group. Given that we did not perform routine stress evaluations in patients with PD, we cannot rule out the possibility that some patients were under a more stressful psychological state in the preoperative MedOff state, which could have exacerbated the tremors and increased the tremor score. Antiparkinsonian medication and DBS surgery might confer a “relaxation effect” on such stress-related tremors. Moreover, the postoperative microlesional effect (Mann et al., 2009) could also contribute to the improvement of tremor score baseline following surgery.

Our findings also indicate that STN-DBS may be superior to GPi-DBS for controlling tremors that contain an L-dopa-resistant component in PD patients. The L-dopa-responsive tremor may be anchored in the dopamine depletion in the nigrostriatal system and exacerbated by beta oscillation in the cortico-basal ganglia-thalamo-cortical circuit, which could be effectively modulated by both GPi- and STN-DBS. Therefore, not surprisingly, the preoperative L-dopa responsiveness and GPi-DBS responsiveness were significantly correlated regarding the tremor subscore of MDS UPDRS-III. On the other hand, the L-dopa-resistant tremor may be due to increased contributions of non-dopaminergic brain regions, such as the cerebellum (Dirkx et al., 2019). Here, we suggest that the dentate-rubro-thalamic tract (DRTT) might be a critical component in the modulation of L-dopa-resistant tremors in PD. The DRTT connects the dentate nucleus in the cerebellum and thalamus, while sending collaterals to the red nucleus (Gallay et al., 2008; Kwon et al., 2011; Petersen et al., 2018). Coenen et al. (2011) reported the case of a patient with therapy-refractory tremor, who was treated by DRTT-DBS. They postulated that the tremor suppression effect of three traditional DBS targets [ventral intermediate nucleus (Vim) (Cury et al., 2017), caudal zona incerta (Blomstedt et al., 2018; Mostofi et al., 2019), and STN] were related to the spread of stimulating effects to DRTT (Coenen et al., 2011). Fiechter et al. (2017) analyzed the mean stimulation site and related volume of tissue activated in patients with tremors undergoing STN- or Vim-DBS, and reported that the DRTT within the posterior subthalamic area might be the common structure stimulated in both cases. Most recently, Coenen et al. (2020) proposed that the DRTT could be a potential common DBS target for tremors of various origins by demonstrating that the latter could be efficiently suppressed by stimulation along the DRTT, despite the variations in tremors etiology. DRTT proximity has also been shown to be associated with lower amplitudes and higher DBS efficiency in essential tremor (Dembek et al., 2020). STN-DBS responsiveness was not correlated with preoperative L-dopa responsiveness in the tremor score in our patients, probably since STN-DBS could effectively suppress not only the L-dopa-responsive tremor by modulating the cortico-basal-ganglia-thalamo-cortical loop, but also the L-dopa-resistant tremor by modulating the adjacent DRTT. The possible association between tremor suppression efficacy of STN-DBS in PD and the distance between electrode contacts and DRTT warrants further investigation.

We also identified age at the time of surgery as a predictor of short-term motor outcomes of STN-DBS with a negative correlation in PD, suggesting that the motor benefit of STN-DBS could be more obscure in elderly PD patients eligible for DBS surgery. GPi-DBS motor responsiveness, however, was insensitive to age. Therefore, a preference of GPi- to STN-DBS in elderly PD patients who have a satisfactory dopamine response could be proposed.

Moreover, we observed a significant postoperative deterioration in the baseline bradykinesia score in patients who underwent GPi, as well as a declining tendency in the STN group, compared to the preoperative MedOff state. This could be attributed to the interplay between disease progression and the underlying insertional, microlesion effects (Mann et al., 2009), and residual effects of DBS (Temperli et al., 2003).

The small sample size, short-term follow-up, non-randomized group allocation, and retrospective design are the main limitations of this study. A more extensive randomized, prospective long-term study is warranted to confirm our findings. Moreover, the evaluation of posture instability and gait disorders in PD was limited, since only the MDS UPDRS-III data were used. Other rating scales that are specifically suited to these aspects should be included in future studies.

## Conclusion

To conclude, our findings suggest that: (1) GPi-DBS would be effective in controlling dopamine-responsive tremors but not dopamine-resistant tremors; (2) the age at the time of surgery may have a negative impact on the motor outcomes of STN-DBS; (3) for patients with dopamine-resistant tremors as a major complaint, STN-DBS may be superior to GPi-DBS; and (4) GPi-DBS could be considered for elderly patients with a satisfactory dopamine response.

## Data Availability Statement

The raw data supporting the conclusions of this article will be made available by the authors, without undue reservation.

## Ethics Statement

The studies involving human participants were reviewed and approved by the Ethical Committee of Ruijin Hospital. The patients/participants provided their written informed consent to participate in this study.

## Author Contributions

DL and PH: study concept and design. YZ and LW: clinical evaluations. ZL and DL: analysis and interpretation of the data. ZL and XZ: drafting of the manuscript. QS, DL, BS, PH, and HZ: critical revision of the manuscript. All authors contributed to the article and approved the submitted version.

## Conflict of Interest

The authors declare that the research was conducted in the absence of any commercial or financial relationships that could be construed as a potential conflict of interest.
