# Corrigendum: Revisiting the L-dopa response as a predictor of motor outcomes after deep brain stimulation in Parkinson's disease

**DOI:** 10.3389/fnhum.2023.1349628

**Published:** 2023-12-15

**Authors:** Zhengyu Lin, Xiaoxiao Zhang, Linbin Wang, Yingying Zhang, Haiyan Zhou, Qingfang Sun, Bomin Sun, Peng Huang, Dianyou Li

**Affiliations:** ^1^Department of Neurosurgery, Ruijin Hospital, Shanghai Jiao Tong University School of Medicine, Shanghai, China; ^2^Center for Functional Neurosurgery, Ruijin Hospital, Shanghai Jiao Tong University School of Medicine, Shanghai, China; ^3^Department of Neurology, Ruijin Hospital, Shanghai Jiao Tong University School of Medicine, Shanghai, China

**Keywords:** Parkinson's disease, L-dopa challenge test, deep brain stimulation, globus pallidus interna, subthalamic nucleus

In the published article, there was an error in the Funding statement. The funding received by PH was not declared in the original published article. The correct Funding statement appears below.

