# Supplementary material for: Revisiting the L-Dopa Response as a Predictor of Motor Outcomes After Deep Brain Stimulation in Parkinson’s Disease
Source: Front Hum Neurosci. 2021 Feb 4;15:604433. doi: 10.3389/fnhum.2021.604433 (PMC7889513; doi:10.3389/fnhum.2021.604433)
Supplement: Supplementary file 1 [file Table_1.DOCX]

**Supplementary Table 1. Stimulating parameters at the last follow-up.**

|  | GPi | STN |
| --- | --- | --- |
| Amplitude (V) |  |  |
| Left | 3.3 ± 0.5 | 2.5 ± 0.5 |
| Right | 3.2 ± 0.5 | 2.5 ± 0.5 |
| Pulse width (µs) |  |  |
| Left | 70.0 ± 8.2 | 60.8 ± 4.9 |
| Right | 69.0 ± 9.9 | 58.5 ± 9.0 |
| Frenquency (Hz) |  |  |
| Left | 143.0 ± 15.5 | 121.5 ± 18.2 |
| Right | 147.5 ± 12.7 | 131.2 ± 22.0 |
